# Imaging cardiac innervation in amyloidosis

**DOI:** 10.1007/s12350-017-1059-9

**Published:** 2017-09-08

**Authors:** Riemer H. J. A. Slart, Andor W. J. M. Glaudemans, Bouke P. C. Hazenberg, Walter Noordzij

**Affiliations:** 1 Department of Nuclear Medicine and Molecular Imaging (EB50), Medical Imaging Center, University Medical Center Groningen, University of Groningen, Hanzeplein 1, P.O. Box 30.001, 9700 RB Groningen, The Netherlands; 2 Department of Rheumatology & Clinical Immunology, University Medical Center Groningen, University of Groningen, Groningen, The Netherlands; 3 Department of Biomedical Photonic Imaging, University of Twente, Enschde, The Netherlands

**Keywords:** Amyloidosis, Sympathetic, Innervation, MIBG

## Abstract

Cardiac amyloidosis is a form of restrictive cardiomyopathy resulting in heart failure and potential risk on arrhythmia, due to amyloid infiltration of the nerve conduction system and the myocardial tissue. The prognosis in this progressive disease is poor, probably due the development of cardiac arrhythmias. Early detection of cardiac sympathetic innervation disturbances has become of major clinical interest, because its occurrence and severity limits the choice of treatment. The use of iodine-123 labelled metaiodobenzylguanidine ([I-123]MIBG), a chemical modified analogue of norepinephrine, is well established in patients with heart failure and plays an important role in evaluation of sympathetic innervation in cardiac amyloidosis. [I-123]MIBG is stored in vesicles in the sympathetic nerve terminals and is not catabolized like norepinephrine. Decreased heart-to-mediastinum ratios on late planar images and increased wash-out rates indicate cardiac sympathetic denervation and are associated with poor prognosis. Single photon emission computed tomography provides additional information and has advantages for evaluating abnormalities in regional distribution in the myocardium. [I-123]MIBG is mainly useful in patients with hereditary and wild-type ATTR cardiac amyloidosis, not in AA and AL amyloidosis. The potential role of positron emission tomography for cardiac sympathetic innervation in amyloidosis has not yet been identified.

**Electronic supplementary material:**

The online version of this article (doi:10.1007/s12350-017-1059-9) contains supplementary material, which is available to authorized users.

## Introduction

Patients with amyloidosis are prone to developing disturbances in autonomic innervation: dysautonomia.^1^ Cardiac dysautonomia can be caused by amyloid infiltration into the myocardial and conduction tissue, resulting in conduction and rhythm disorders. Cardiac dysautonomia is common in patients with transthyretin-related amyloidosis (ATTR type) and in patients with immunoglobulin light chain-derived amyloidosis (AL type).^2^ More specific, patients with the hereditary form of ATTR type amyloidosis (hATTR, formerly called familial amyloid polyneuropathy) frequently develop polyneuropathy and dysautonomia. Furthermore, cardiac dysautonomia may occur independent of the presence of a typical restrictive cardiomyopathy. Amyloidosis’ typical restrictive cardiomyopathy is most commonly found in patients with wild-type ATTR type amyloidosis (wtATTR, formerly called senile systemic amyloidosis). In these wtATTR patients, polyneuropathy and dysautonomia are infrequent and approximately 9%.^3^

At present, actual amyloid infiltration cannot be visualized with nuclear medicine techniques. Nonetheless, semi-quantitative analysis of tracer accumulation in the left ventricle compared to the background (heart-to-mediastinum ratio, HMR) on iodine-123 labelled metaiodobenzylguanidine ([I-123]MIBG) scintigraphy, is assumed to provide insight in the amyloid infiltration of the sympathetic nerve system.^4^–^12^ [I-123]MIBG, a chemically modified analogue of norepinephrine, is stored in vesicles in presynaptic sympathetic nerve terminals and not further catabolized. Decreased HMR at 4 h after tracer administration (late HMR) reflects the degree of sympathetical dystonia, and is found to be an independent prognostic factor in the development of ventricular dysrhythmia.^13^ Whereas showing promising results in ischemic heart disease, positron emission tomography (PET) for sympathetic innervation in cardiac manifestation of amyloidosis has not yet been studied.^14^

**Figure 1:**
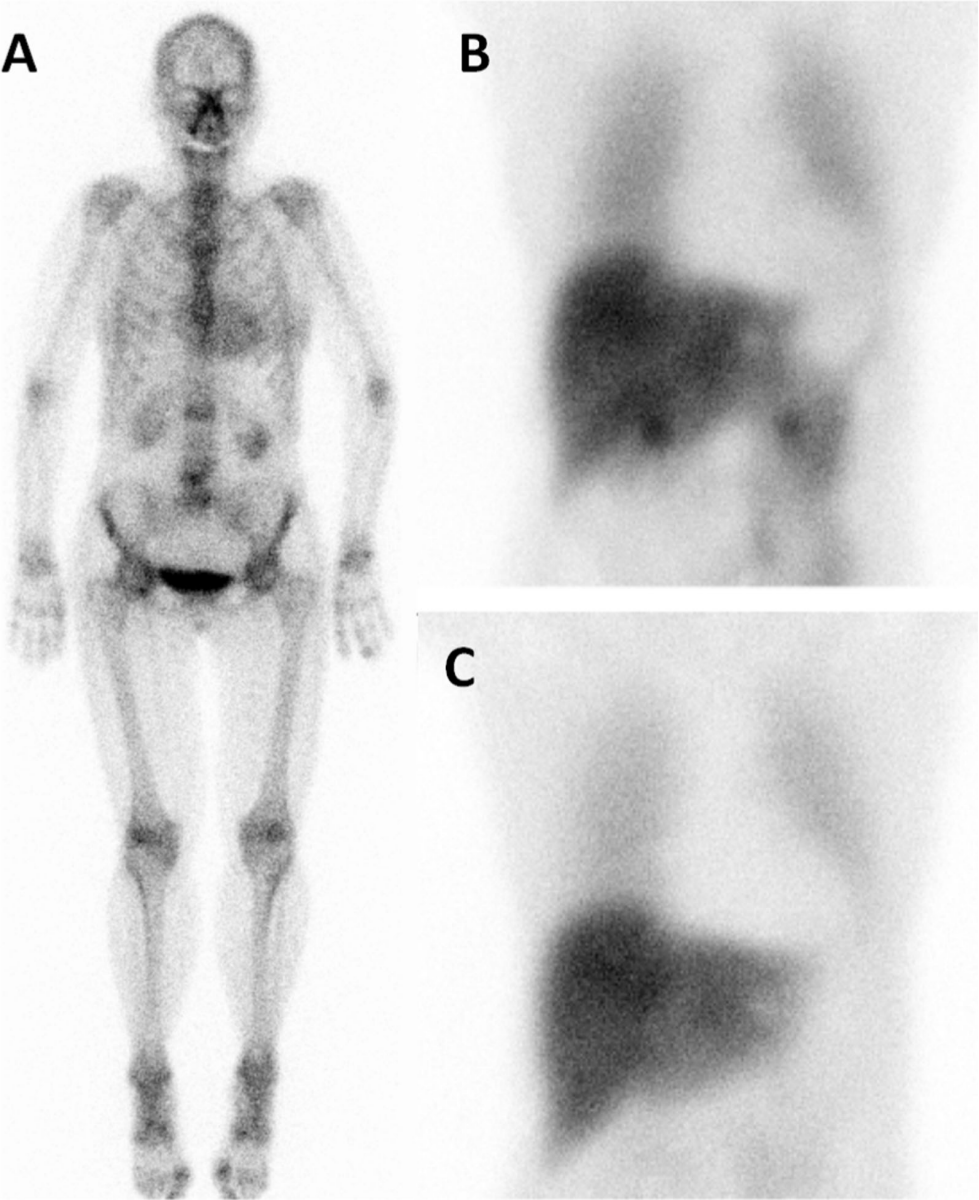
Example of a 70 year old female patient with ATTR amyloidosis based on Val30Met mutation, with both positive bone scan (**A**) and [I-123]-MIBG scintigraphy. **B** 15 minutes post injection (p.i.), **C** 4 hours p.i.. Late HMR 1.38, normal value in our laboratory: 2.0, performed with a medium energy collimator

**Figure 2:**
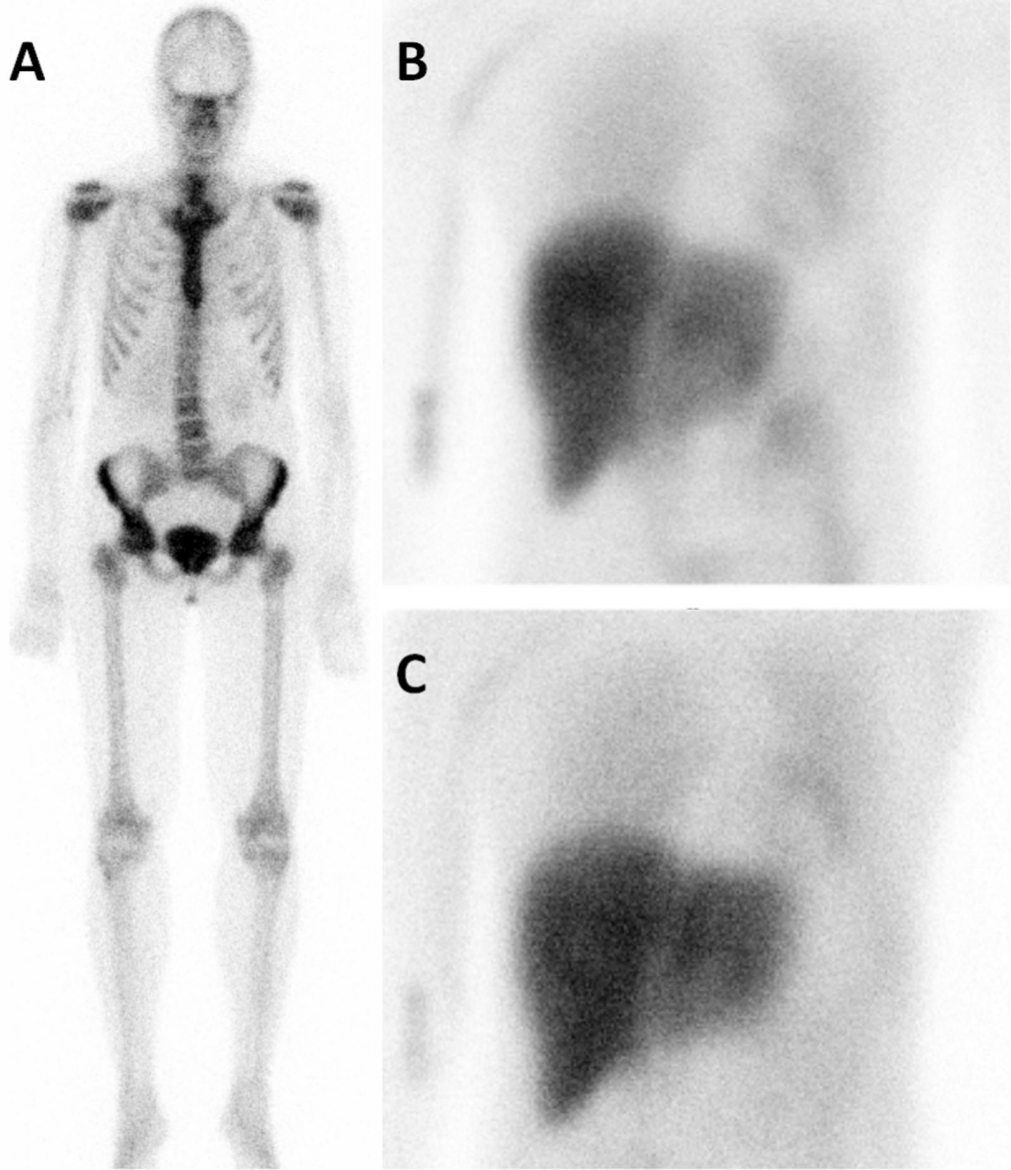
Example of a 42 year old female patients with hereditary ATTR amyloidosis (TTR-Tyr114Cys), without cardiac bone tracer accumulation (**A**), but impaired cardiac sympathetic innervation (**B** 15 minutes p.i., **C** 4 hours p.i.). Late HMR 1.63

**Figure 3:**
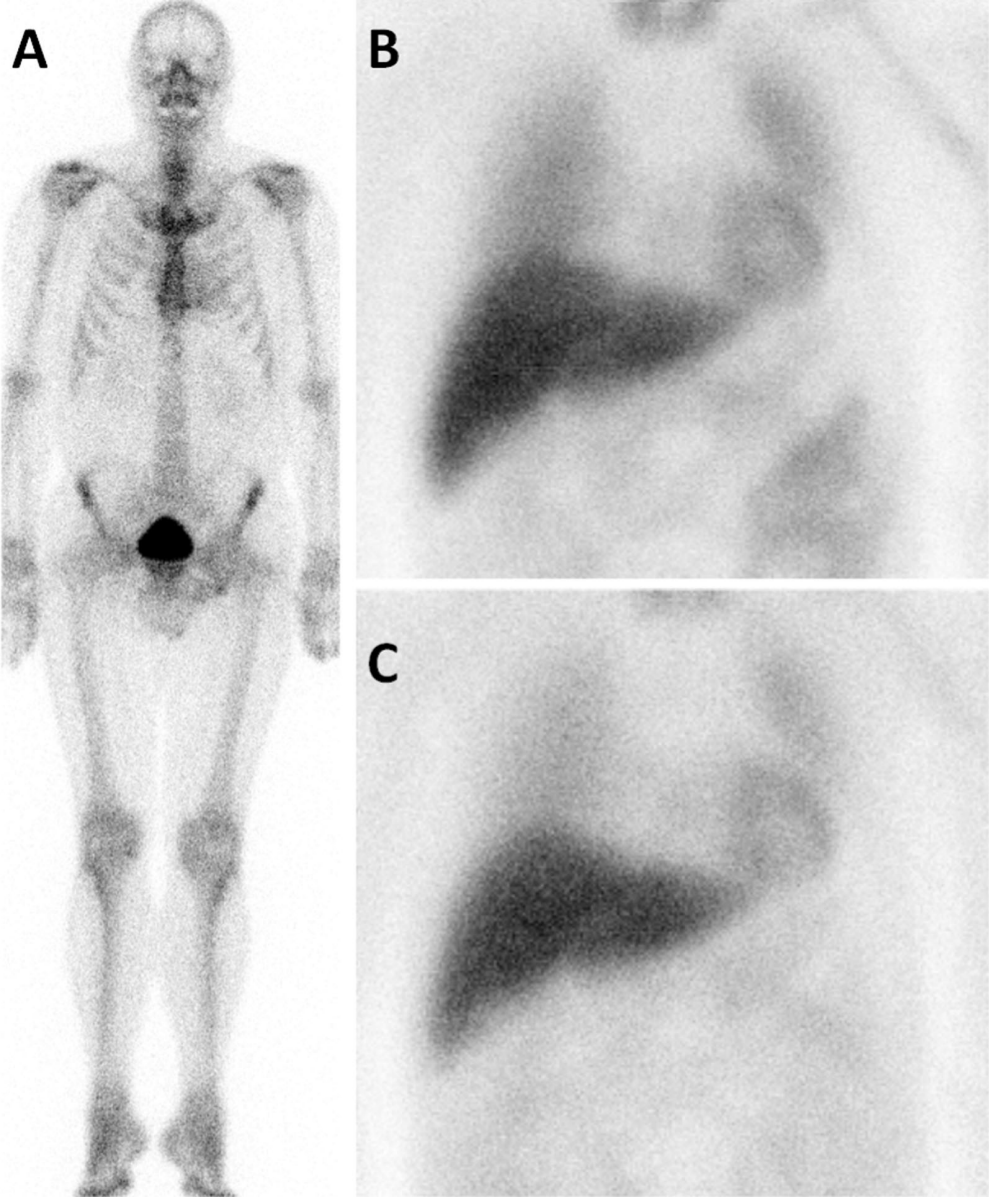
Example of a 60 year old male patients with ATTR amyloidosis based on Val50Met mutation, with slightly elevated cardiac bone tracer accumulation (**A**), but [I-123]-MIBG scintigraphy within normal ranges (**B** 15 minutes p.i., **C** 4 hours p.i.). Late HMR 2.2

The purpose of this review is to provide an overview of the present literature on the application of nuclear imaging modalities for the evaluation of cardiac innervation in patients with amyloidosis, and its future perspectives (Figures 1, 2, 3).

## Methods

For this review a literature search was performed on PubMed on May 5th 2017, using the following string: {[(innervation) OR (sympathetic)] AND [(amyloidosis) OR (amyloid)] AND [(heart) OR (cardiac)] AND [(nuclear) OR (imaging)]}, resulting in 29 hits, of which 24 were considered relevant for this review. Reviews, editorials, abstracts, case reports, animal studies, conference presentations were excluded. In total, 16 articles were found that used radiopharmaceuticals for conventional nuclear medicine imaging of cardiac innervation, all with [I-123]MIBG. The results of these papers are summarized below and divided into three main topics: the imaging of cardiac innervation itself, the implications of this imaging method, and the relation with other nuclear medicine imaging techniques in cardiac amyloidosis.

## Imaging of Cardiac Innervation in Amyloidosis

Imaging of cardiac innervation in patients with amyloidosis has been mainly focused on visualizing the effects of amyloidosis on the sympathetic nerve system. Conventional nuclear imaging [I-123]MIBG is the most widely used modality for this indication. Table 1 provides an overview of the present available literature with respect to the use of [I-123]MIBG in patients with different types of systemic amyloidosis. The main results regarding HMR, wash-out and patient outcome of the different studies are displayed. As shown in this overview, hATTR type amyloidosis patients are studied most extensively, showing the most pronounced reduced late HMR. Also AL type amyloidosis patients tend to have decreased late HMR compared to healthy control subjects, however to a lesser extent compared to both hATTR and wtATTR type patients.^8^,^10^,^12^ Due to the large overlap of late HMR ranges in ATTR and AL type amyloidosis patients, [I-123]MIBG scintigraphy is considered not to be able to discriminate between these amyloidosis subtypes.^12^ Despite that cardiac manifestations are very rare in patients with secondary (AA) amyloidosis, one study showed lower mean late HMR in 11 AA type amyloidosis patients compared to healthy control subjects.^12^ This finding may contribute to the assumption of amyloid deposits infiltrating the conducting system during the course of the disease.

**Table 1:** Main results and patient outcome as reported in studies using Iodine-123 labelled metaiodobenzylguanidine scintigraphy in patients with amyloidosis

Study	Author, year of publication	Number of patients	Tracer dose	Collimator type	Time point late HMR	Amyloid typing	Main results	Patient outcome
1	Nakata et al^4^	1 patient	111 MBq (3 mCi) [I-123]-MIBG	N/A	4 hours p.i.	hATTR (TTR Val30Met)	No cardiac tracer accumulation	N/A
2	Tanaka et al^5^	12 patients	148 MBq (4 mCi) [I-123]-MIBG	LE	3 hours p.i.	hATTR	No cardiac tracer accumulation in 8 of 12	Mean FU 15.5 ± 5.8 months: no lethal arrhythmia, no cardiac death
3	Delahaye et al^6^	17 patients, 12 healthy controls	300 MBq (8 mCi) [I-123]-MIBG	LE	4 hours p.i.	hATTR	Mean late HMR in patients 1.36 ± 0.26 vs in healthy controls 1.98 ± 0.35 (*P* < 0.001), no difference in wash-out	N/A
4	Delahaye et al^35^	21 patients, 12 healthy controls	150 and 180 MBq (4 and 5 mCi) [C-11]-MQNB and 300 MBq (8 mCi) [I-123]-MIBG	LE	4 hours p.i.	hATTR (20 patients TTR Val30Met, 1 patient TTR Thr49Ala)	Mean muscarinic receptor density was higher in patients than in control subjects: B’max, 35.5 ± 8.9 vs 26.1 ± 6.7 pmol/mL (*P* *=* 0.003) Mean late HMR in patients 1.43 ± 0.28 vs in healthy controls 1.98 ± 0.35 (*P* < 0.001), mean wash-out 29% ± 6.8% vs 21% ± 6% (*P* = 0.003). Individual muscarinic receptor density did not correlate with late HMR	N/A
5	Watanabe et al^9^	4 patients, 10 age-matched controls	111 MBq (3 mCi) [I-123]-MIBG	N/A	4 hours p.i.	hATTR (TTR Val30Met)	Mean late HMR in patients 1.1 ± 0.2, vs 2.4 ± 0.2 in health controls (p-value N/A)	N/A
6	Hongo et al^8^	25 patients, of which 16 patients without and 9 patients with autonomic neuropathy	111 MBq (3 mCi) [I-123]-MIBG	LE	3 hours p.i.	AL	Mean late HMR in patients without autonomic neuropathy 1.53 ± 0.06 vs in with autonomic neuropathy 1.29 ± 0.05 (*P* < 0.001), mean wash-out 42 ± 4.8% vs 31 ± 4.0% (*P* < 0.001)	N/A
7	Lekakis et al^10^	3 patients, 23 controls	185 MBq (5 mCi) [I-123]-MIBG	LE	4 hours p.i.	AL	Mean late HMR 1.33 ± 0.1 vs in 2.13 ± 0.2 healthy controls (*P* value N/A)	N/A
8	Coutinho et al^28^	34 patients, of which 2 patients without and 12 patients with autonomic neuropathy	[I-123]-MIBG (dose N/A)	N/A	N/A	hATTR	Mean late HMR 1.75 ± 0.5 in all patients. Mean late HMR in patients without neuropathy 2.2 ± 0.5 vs patients with neuropathy 1.5 ± 0.4 (*P* = 0.001)	N/A
9	Delahaye et al^11^	31 patients	300 MBq (8 mCi) [I-123]-MIBG	LE	4 hours p.i.	hATTR	Mean late HMR 2 years after liver transplantation 1.46 ± 0.28 vs 6 months before liver transplantation 1.45 ± 0.29, *P* = not significant	No cardiac death or lethal arrhythmia reported
10	Algalarrando et al^36^	32 patients	300 MBq (8 mCi) [I-123]-MIBG	LE	4 hours p.i.	hATTR	Late HMR ≤1.6 in 26 out of 32 patients	No cardiac death or lethal arrhythmia reported
11	Noordzij et al^12^	61 patients, 9 healthy control subjects	185 MBq (5 mCi) [I-123]-MIBG	ME	4 hours p.i.	AL (39 patients), AA (11 patients), ATTR (11 patients)	Mean late HMR in all patients 2.3 ± 0.75 vs healthy control subjects 2.9 ± 0.58 (*P* < 0.005). Mean late HMR in ATTR patients 1.7 ± 0.75 vs AL patients 2.4 ± 0.75 (*P* < 0.05). Mean wash-out in patients 8.6% ± 14% vs in healthy control subjects −2.1% ± 10% (*P* < 0.05)	No cardiac death or lethal arrhythmia
12	Noordzij et al^37^	2 patients	185 MBq (5 mCi) [I-123]-MIBG	ME	4 hours p.i.	wtATTR, hATTR (TTR Val122Ile)	Patient A: late HMR 1.57, wash-out >20%, patient B: late HMR 1.13, wash-out 28%	N/A
13	Coutinho et al^21^	143 patients	185 MBq (5 mCi) [I-123]-MIBG	LE	3 hours p.i.	hATTR (TTR Val30Met)	Mean late HMR 1.83±0.43, and mean was-out 47±11%	Mean FU 5.5 years: hazard ratio all-cause mortality 7 if HMR <1.6, progressive increase in 5-year mortality with decrease in late HMR
14	Takahashi et al^38^	6 patients	[I-123]-MIBG (dose N/A)	N/A	N/A	hATTR (TTR Val30Met)	Mean late HMR at baseline 1.7 ± 0.9 vs after 3 year diflunisal treatment 1.9 ± 1.0 (*P* = 0.004). Mean wash-out at baseline 46% ± 20% vs after 3 years 43% ± 23% (*P* = 0.67)	No cardiac death or lethal arrhythmia reported
15	Algalarrando et al^22^	215 patients	3 MBq/kg (0.08 mCi/kg) [I-123]-MIBG	LE	4 hours p.i.	hATTR (148 patients TTR Val30Met)	Median late HMR 1.49 (Inter-quartile range 1.24–1.74, range 0.97–2.52)	Median FU 5.9 years after liver transplantation: 5-year survival 64% if late HMR ≤1.43, vs 93% if HMR >1.43 (*P* < 0.0001)
16	Azevedo Coutinho et al^23^	232 patients	185 MBq (5 mCi) [I-123]-MIBG	LE	3 hours p.i.	hATTR (TTR Val30Met)	Initial assessment: mean late HMR 1.83 ± 0.03, median wash-out 2.5 (Inter-quartile range −2.3–8.5) During follow-up late HMR decreased with age and duration of neurological symptoms, but stabilized after liver transplantation. Mean late HMR at inclusion was higher in patients who were still alive at the end of FU, compared to those who deceased: 1.90 ± 0.37 vs 1.58 ± 0.40, *P* < 0.001	Median FU 4.5 years (inter-quartile range 2.1–7.7 years). Initial HMR <1.55: HR mortality 9.36 (95% CI 4.27–20.56, *P* < 0.001) Initial HMR 1.55–1.83: HR mortality 4.27 (95% CI 1.68–9.05, *P* = 0.002)

*[I-123]-MIBG*, Iodine-123 labelled metaiodobenzylguanidine; *[C-11]-MQNB*, carbon-11 labelled methylquinuclidinyl benzilate; *Hattr*, hereditary transthyretin-derived amyloid; *wtATTR*, wild-type transthyretin-derived amyloid; *AL*, immunoglobulin light chain-derived amyloid; *FU*, follow-up; *HMR*, heart-to-mediastinum ratio; *HR*, hazard ratio; *LE*, low energy; *ME*, medium energy; *N/A*, not available; *p.i.*, post injection

Mean late HMR differs substantially between the different publications. This variability is mainly due to non-homogeneity in [I-123]MIBG imaging acquisition. HMR varies between different gamma camera systems (venders), but more importantly between the application of low energy and medium energy collimators.^15^ Generally, HMR is higher on images acquired with medium energy collimators compared to images acquired with low energy collimator.^16^ Based on these differences in HMR, cut-off values for the different collimators are proposed, as well as conversion algorithms.^17^,^18^

Additional single photon emission computed tomography (SPECT) scanning may be of value in the evaluation of regional cardiac sympathetic innervation abnormalities. The majority of patients (both AL and ATTR type amyloidosis) with low HMR show reduced tracer accumulation in the infero-postero-lateral segments.^4^–^8^,^11^ Unfortunately, this may not be considered as a characteristics finding in amyloidosis patients, since a defect in [I-123]MIBG accumulation in the inferior myocardial wall is also reported in healthy control subjects.^19^ This is considered as a consequence of physiological [I-123]MIBG accumulation in the liver overprojecting the infero-posterior myocardial wall.

## Implications of Impaired Cardiac Sympathetic Innervation

Studies using [I-123]MIBG in patients with ischemic heart disease (IHD) have shown that disrupted cardiac sympathetic innervation based on low late HMR is associated with an increased risk on developing ventricular arrhythmia and appropriate implantable cardioverter-defibrillator (ICD) shocks, and is associated with poor survival.^13^,^20^ In fact, reduced late HMR is a stronger prognostic factor than left ventricular ejection fraction (LVEF) for developing severe adverse cardiac events in patients with IHD.^13^ In amyloidosis patients with impaired cardiac sympathetic innervation, decreased survival rates are also established.^21^–^23^ Late HMR was identified as an independent prognostic factor for 5-year all-cause mortality, with a 42% mortality rate for those patients with late HMR <1.60, compared to merely 7% in patients with late HMR ≥1.60 (hazard ratio (HR) 7.2, *P* < 0.001).^21^ Based on the results of this study, even patients with HMR <1.60 seem to benefit from liver transplantation (because of amyloid involvement), resulting in lower long-term mortality than neurophysiological score-matched control subjects (HR 0.32, *P* = 0.012).^21^ This underlines the assumption that impaired cardiac sympathetic innervation will not progress after liver transplantation, and that re-innervation cannot be detected within this duration of clinical follow-up.^11^,^23^

In addition, late HMR remains of prognostic importance after liver transplantation, with larger area under the receiver-operating curve than clinical parameters and heart rate variability (AUC: 0.79 vs 0.66 and 0.52, respectively) in univariate analysis.^22^ However, multivariate analysis revealed that late HMR has no additive value to a reference model in predicting outcome (AUC 0.80 vs 0.79, respectively).^22^

In the AL type population, very little is known about the consequences of reduced late HMR. Follow-up of the available studies in this population is too limited to identify arrhythmogenic consequences of impaired cardiac sympathetic innervation.^8^,^10^,^12^

Data on the contribution of reduced late HMR to cardiovascular outcome measurements in patients with ATTR amyloidosis seems to be incomplete. Only one study reported the association of reduced late HMR with the presence of ventricular arrhythmia, and the progression of conduction disturbances after liver transplantation due to continuous amyloid infiltration.^11^ Understanding this apparent oxymoron (i.e.: the cessation of progression of cardiac innervation abnormalities despite continuous amyloid infiltration after liver transplantation) will be a challenge for future investigations. As of yet, the actual incidence of ventricular arrhythmia, sudden cardiac death, or appropriate ICD shocks in amyloidosis patients with impaired cardiac sympathetic innervation is not fully elucidated. Therefore, the question whether amyloidosis patients will benefit from prophylactic ICD remains unanswered.

## Relation to Other Nuclear Imaging Modalities in Amyloidosis

In early studies using [I-123]MIBG, amyloidosis patients underwent additional (rest) myocardial perfusion scintigraphy using thallium-201 ([Tl-201]).^4^–^7^,^9^–^11^ None of the included patients seemed to suffer from myocardial infarction, since all rest [Tl-201] scans were reported normal, without perfusion defects. This perfusion – innervation mismatch is a known phenomenon in patients with ischemic cardiomyopathy, but also occurs in patients with non-ischemic (dilating) cardiomyopathy.^24^,^25^ Myocardial perfusion abnormalities are known to result in damaged sympathetic nerve terminals, leading to a larger area of impaired innervation than impaired perfusion alone. This mismatch pattern leads to electrophysiological imbalance, which is associated with a higher risk of developing ventricular dysrhythmia.^24^,^25^ The mechanism behind the development of perfusion—innervation mismatch pattern in patients with non-ischemic cardiomyopathy is not fully elucidated. However, the presence of structural changes (for example heterogeneous interstitial fibrosis) may contribute to altered ventricular activation and contractility, due to maladaptation to myocardial injury. In combination with disturbed sympathetic stimulation due to amyloid infiltration, this may contribute to a higher risk of ventricular dysrhythmia in amyloidosis patients as well.

The mutual contribution of autonomic neuropathy and cardiomyopathy to each other on decreased late HMR remains a conundrum. Since both wtATTR and hATTR type amyloidosis patients show decreased late HMR, [I-123]MIBG scintigraphy alone may not be sufficient to discriminate between autonomic neuropathy and cardiomyopathy. Several studies have shown that myocardial bone tracer accumulation discriminates ATTR from AL type amyloidosis.^26^,^27^ Bone tracer accumulation predominantly occurs in wild-type ATTR type patients, probably as a result of the underlying cardiomyopathy. On the contrary, patients with hATTR type amyloidosis without cardiomyopathy tend to show no myocardial bone tracer accumulation, and normal biomarkers (N-terminus pro-brain natriuretic peptide, and troponine-T). Within these patients, late HMR is generally lower in the subgroup of patients with other symptoms of polyneuropathy.^28^ Future studies should focus on the possible additive value of bone scintigraphy in relation to [I-123]MIBG scintigraphy in getting a better understanding of the mutual contribution of neuropathy and cardiomyopathy to each other in ATTR type patients.

Recently in positron emission tomography (PET), carbon-11 labelled Pittsburgh compound-B ([C-11]-PiB), derived from the amyloid stain thioflavin, as well as fluorine-18 ([F-18]) labelled florbetapir have been used as tracers for cardiac amyloid.^29^,^30^ However, their role against cardiac sympathetic innervation is to be determined. There is no role for [F-18] fluorodeoxyglucose (FDG) imaging or [I-123]SAP scintigraphy in evaluating cardiac manifestation against sympathetic innervation disturbances in amyloidosis, since neither one of both tracers is known to accumulate in cardiac amyloid deposits.^31^,^32^

## Future Developments

There is an increasing evidence for the prognostic value of [I-123]MIBG scintigraphy in patients with amyloidosis. However, more prospectively acquired data is needed to implement [I-123]MIBG scintigraphy in guidelines as a standard imaging procedure in the management of (especially ATTR type) amyloidosis patients. Therefore, consensus in acquisition parameters in different study protocols is pivotal. Standardization of collimator choice, imaging acquisition, and data analysis in different studies, is necessary for successful implementation in daily patient practice.^15^

Finally, the use of PET tracers has advantages over [I-123]MIBG in cardiac sympathetic innervation imaging. Carbon-11 labelled meta-hydroxy-ephedrine [C-11]mHED has been extensively studied in patients with both ischemic and non-ischemic cardiomyopathies.^14^,^20^ Based on the studies in patients with left ventricular dysfunction, [C-11]mHED outperforms [I-123]MIBG in detecting regional impaired sympathetic innervation, due to better resolution and absolute quantification.^33^ Despite that [C-11]mHED is the most used PET tracer for visualization of cardiac sympathetic innervation abnormalities, it’s value has not yet been studied in amyloidosis patients. Future studies should provide information on the value of recently developed PET tracers in evaluating cardiac sympathetic innervation in amyloidosis patients. In theory, two new PET tracers may have additional value over [I-123]MIBG scintigraphy in regard to higher HMR. For example, [I-124]MIBG may provide superior image quality, whereas N-[3-Bromo-4-3-[F-18]fluoro-propoxy)-benzyl]-guanidine ([F-18]LM1195) has the additional advantage that an on-site cyclotron is not necessary.^34^

## Conclusions

[I-123]MIBG is currently the most widely used radiopharmaceutical for imaging cardiac sympathetic innervation disturbances in patients with cardiac manifestations of amyloidosis. Particular patients with hATTR type amyloidosis show diminished late HMR’s, and consequently have a higher risk of cardiac mortality.

Future studies should provide better insight into the presence and degree of overlap between cardiac neuropathy and cardiomyopathy in patients with cardiac manifestations of amyloidosis, the role of nuclear medicine modalities in distinguishing cardiac neuropathy from cardiomyopathy, and finally, the potential role of PET tracers in evaluating impaired cardiac sympathetic innervation.

## Electronic supplementary material

Below is the link to the electronic supplementary material.

Supplementary material 1 (PPTX 2061 kb)
