# Supplementary material for: Imaging cardiac innervation in amyloidosis
Source: J Nucl Cardiol. 2017 Sep 8;26(1):174–87. doi: 10.1007/s12350-017-1059-9 (PMC6394628; doi:10.1007/s12350-017-1059-9)
Supplement: Supplementary file 1 — Supplementary material 1 (PPTX 2061 kb) [file 12350_2017_1059_MOESM1_ESM.pptx]

## Slide 1
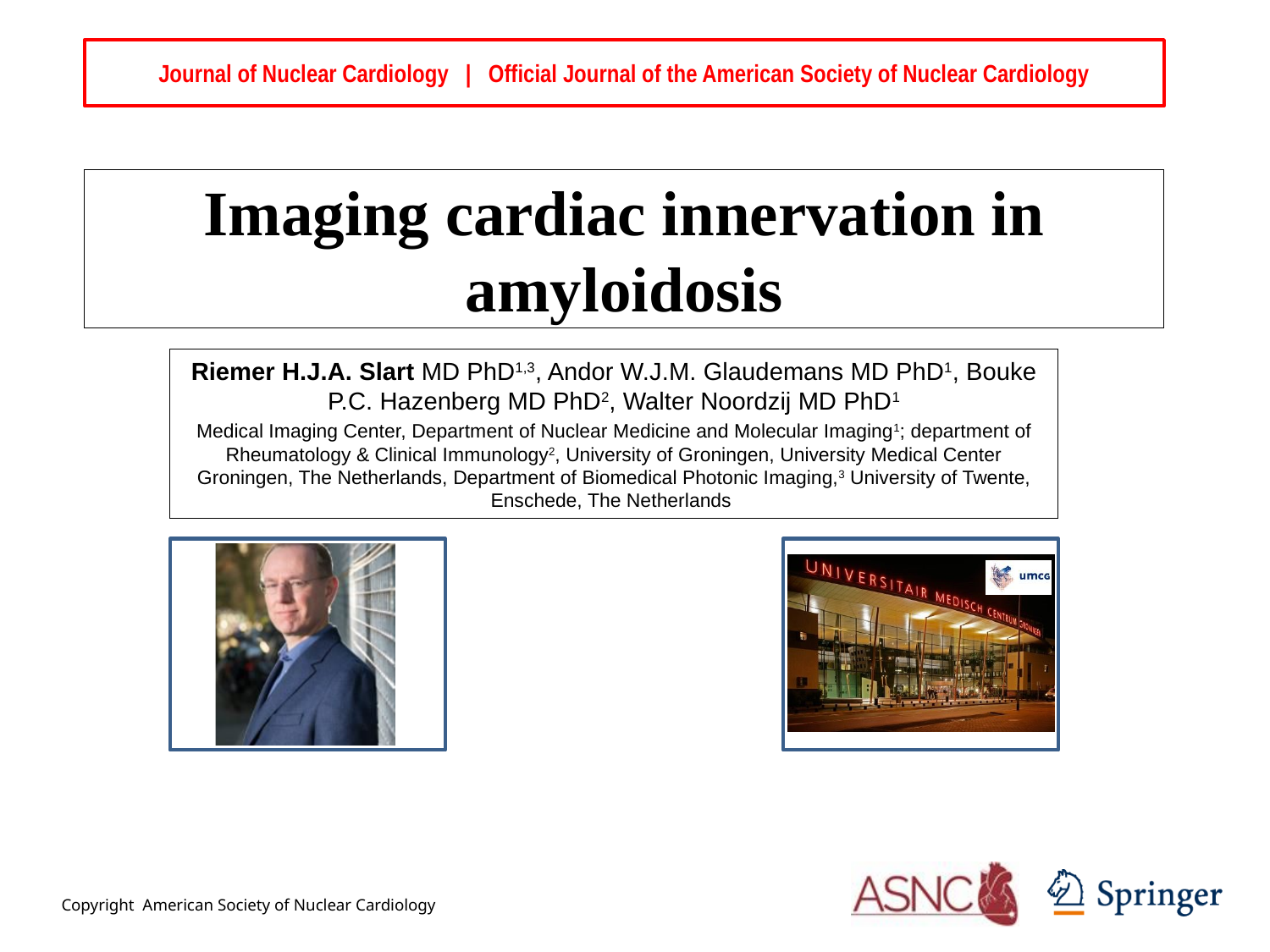

Journal of Nuclear Cardiology | Official Journal of the American Society of Nuclear Cardiology
# Imaging cardiac innervation in amyloidosis
Riemer H.J.A. Slart MD PhD1,3, Andor W.J.M. Glaudemans MD PhD1, Bouke P.C. Hazenberg MD PhD2, Walter Noordzij MD PhD1
Medical Imaging Center, Department of Nuclear Medicine and Molecular Imaging1; department of Rheumatology & Clinical Immunology2, University of Groningen, University Medical Center Groningen, The Netherlands, Department of Biomedical Photonic Imaging,3 University of Twente, Enschede, The Netherlands
Copyright American Society of Nuclear Cardiology

## Slide 2
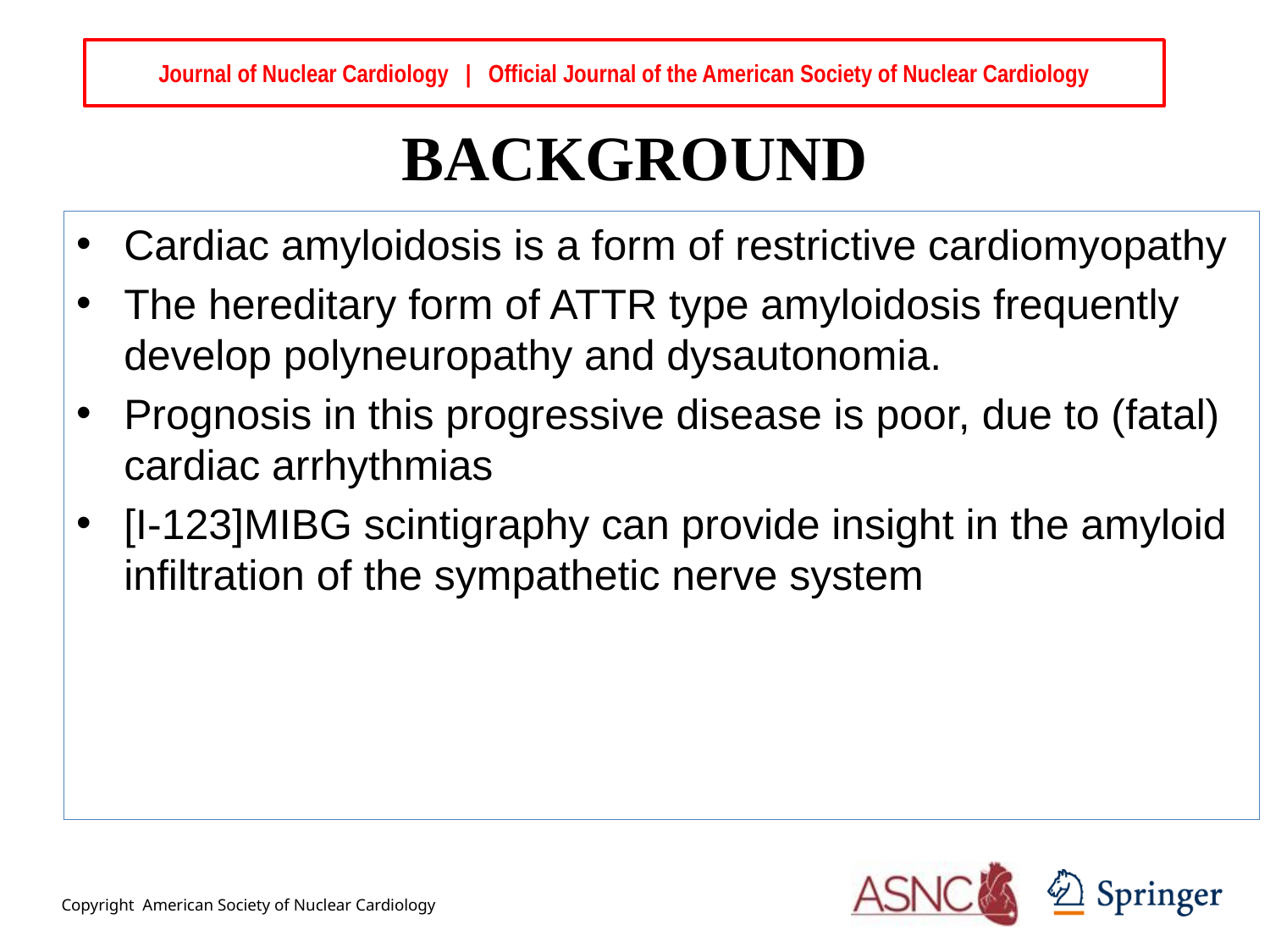

Journal of Nuclear Cardiology | Official Journal of the American Society of Nuclear Cardiology
# BACKGROUND
Cardiac amyloidosis is a form of restrictive cardiomyopathy
The hereditary form of ATTR type amyloidosis frequently develop polyneuropathy and dysautonomia.
Prognosis in this progressive disease is poor, due to (fatal) cardiac arrhythmias
[I-123]MIBG scintigraphy can provide insight in the amyloid infiltration of the sympathetic nerve system
Copyright American Society of Nuclear Cardiology

## Slide 3
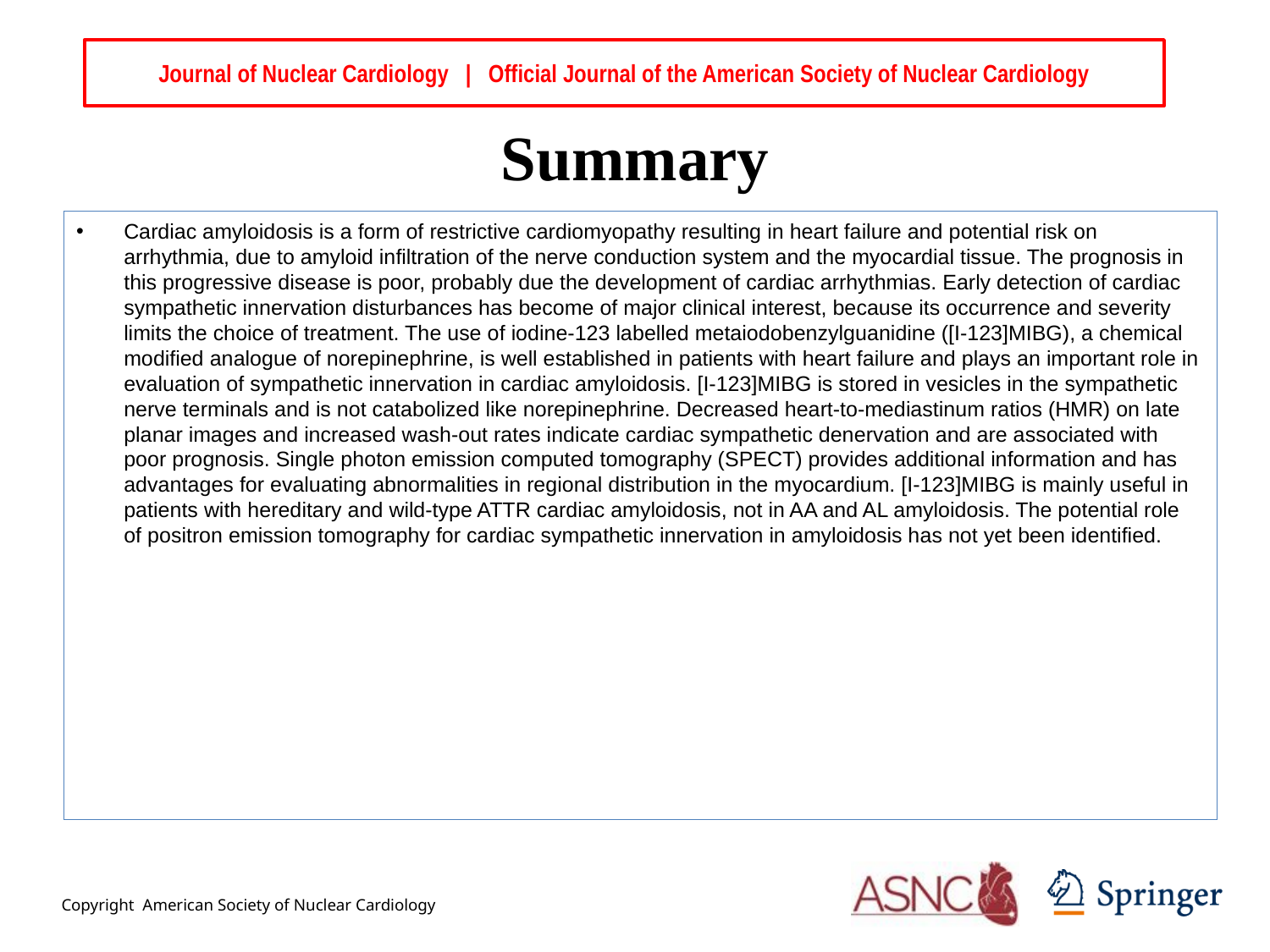

Journal of Nuclear Cardiology | Official Journal of the American Society of Nuclear Cardiology
# Summary
Cardiac amyloidosis is a form of restrictive cardiomyopathy resulting in heart failure and potential risk on arrhythmia, due to amyloid infiltration of the nerve conduction system and the myocardial tissue. The prognosis in this progressive disease is poor, probably due the development of cardiac arrhythmias. Early detection of cardiac sympathetic innervation disturbances has become of major clinical interest, because its occurrence and severity limits the choice of treatment. The use of iodine-123 labelled metaiodobenzylguanidine ([I-123]MIBG), a chemical modified analogue of norepinephrine, is well established in patients with heart failure and plays an important role in evaluation of sympathetic innervation in cardiac amyloidosis. [I-123]MIBG is stored in vesicles in the sympathetic nerve terminals and is not catabolized like norepinephrine. Decreased heart-to-mediastinum ratios (HMR) on late planar images and increased wash-out rates indicate cardiac sympathetic denervation and are associated with poor prognosis. Single photon emission computed tomography (SPECT) provides additional information and has advantages for evaluating abnormalities in regional distribution in the myocardium. [I-123]MIBG is mainly useful in patients with hereditary and wild-type ATTR cardiac amyloidosis, not in AA and AL amyloidosis. The potential role of positron emission tomography for cardiac sympathetic innervation in amyloidosis has not yet been identified.
Copyright American Society of Nuclear Cardiology

## Slide 4
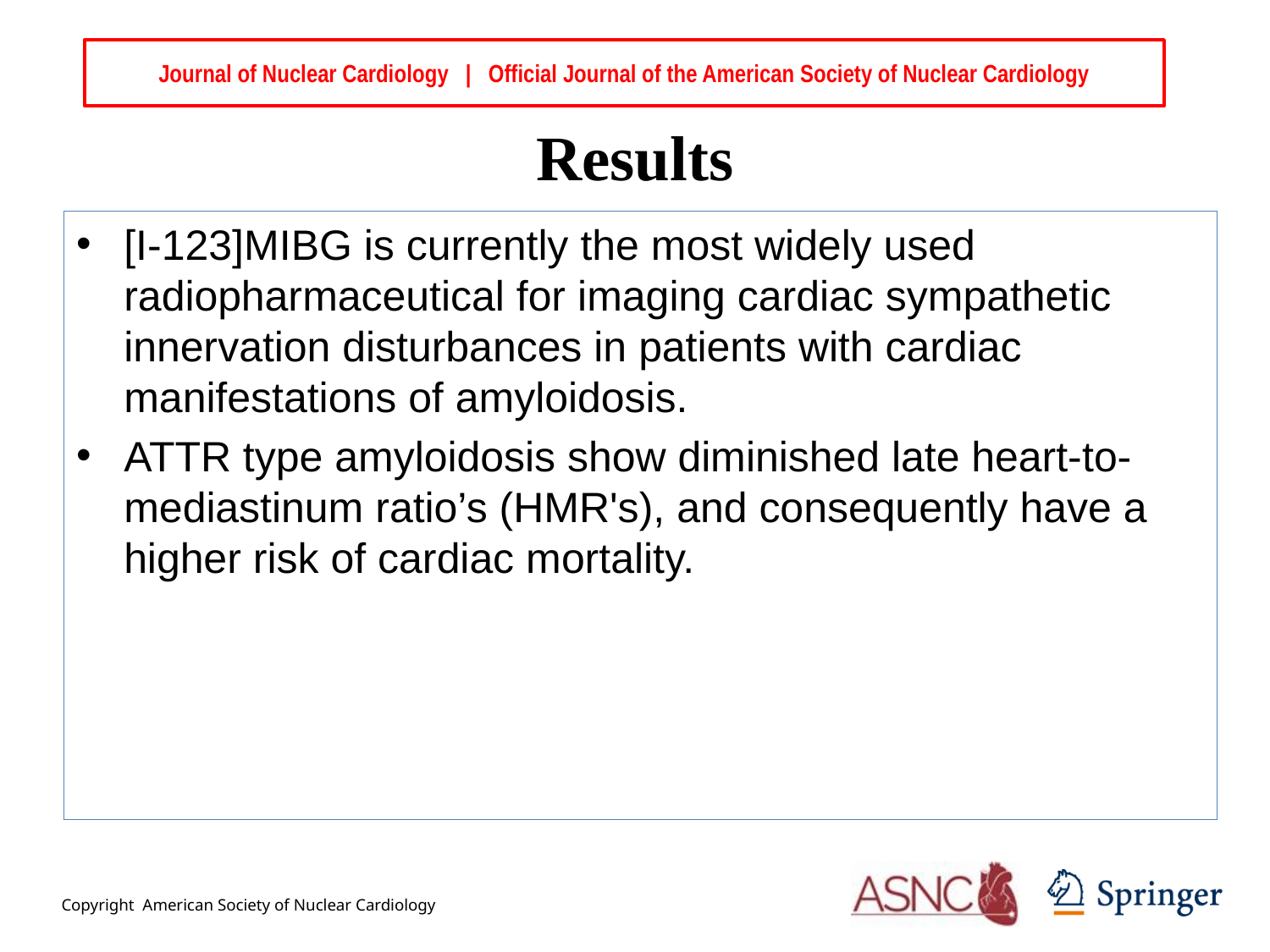

Journal of Nuclear Cardiology | Official Journal of the American Society of Nuclear Cardiology
# Results
[I-123]MIBG is currently the most widely used radiopharmaceutical for imaging cardiac sympathetic innervation disturbances in patients with cardiac manifestations of amyloidosis.
ATTR type amyloidosis show diminished late heart-to-mediastinum ratio’s (HMR's), and consequently have a higher risk of cardiac mortality.
Copyright American Society of Nuclear Cardiology

## Slide 5
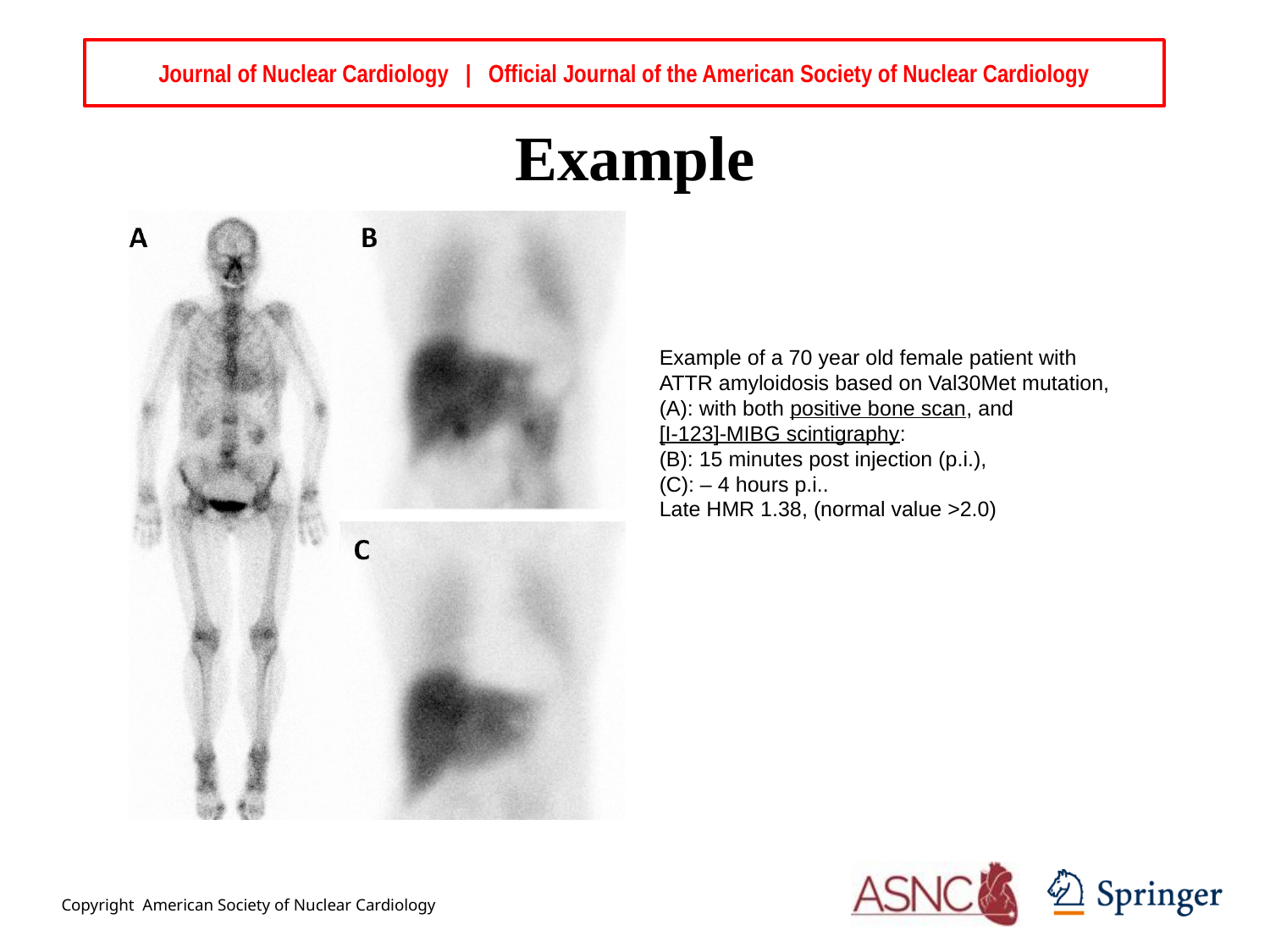

Journal of Nuclear Cardiology | Official Journal of the American Society of Nuclear Cardiology
# Example
Example of a 70 year old female patient with
ATTR amyloidosis based on Val30Met mutation,
(A): with both positive bone scan, and
[I-123]-MIBG scintigraphy:
(B): 15 minutes post injection (p.i.),
(C): – 4 hours p.i..
Late HMR 1.38, (normal value >2.0)
Copyright American Society of Nuclear Cardiology

## Slide 6
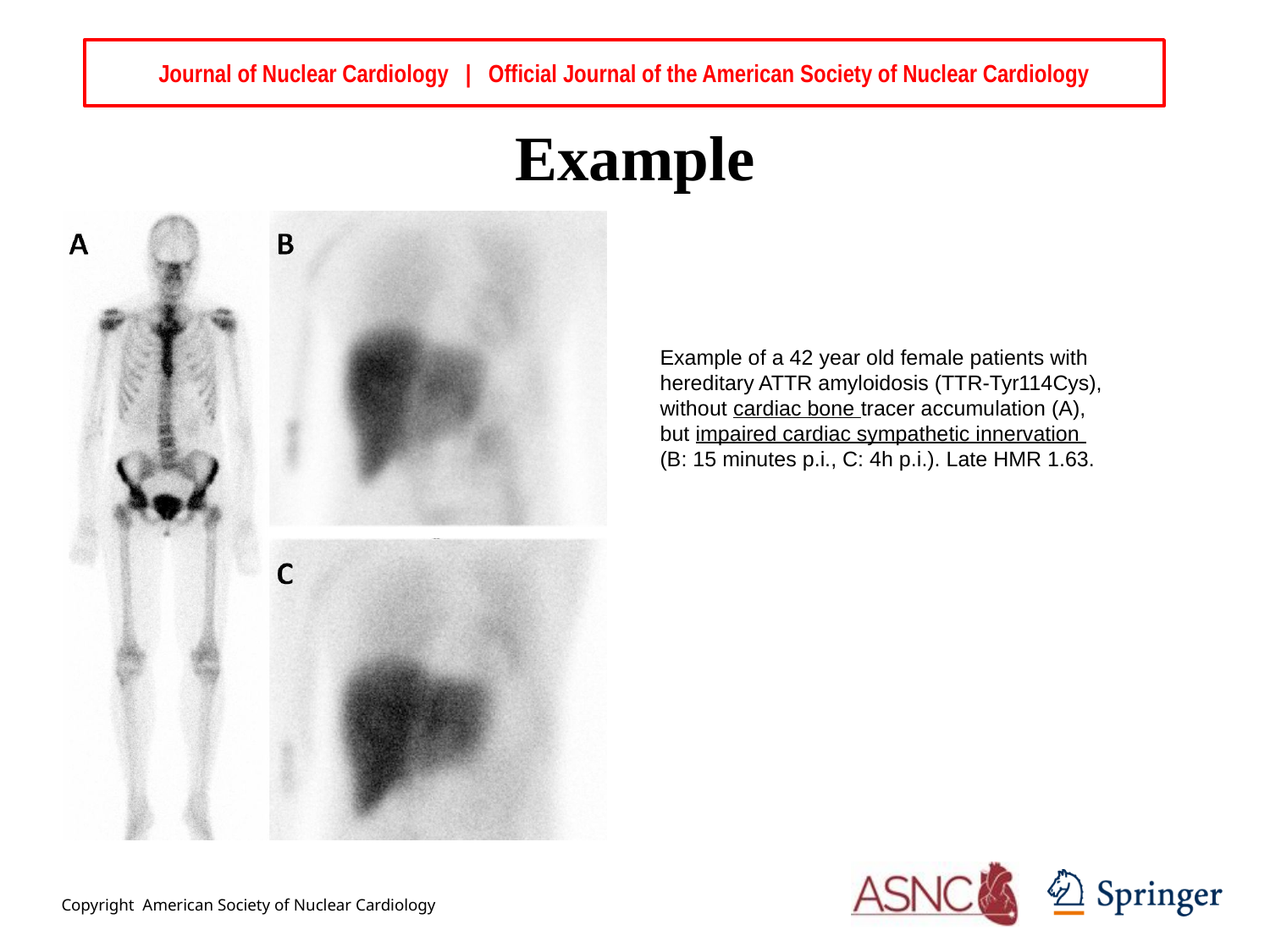

Journal of Nuclear Cardiology | Official Journal of the American Society of Nuclear Cardiology
# Example
Example of a 42 year old female patients with
hereditary ATTR amyloidosis (TTR-Tyr114Cys),
without cardiac bone tracer accumulation (A),
but impaired cardiac sympathetic innervation
(B: 15 minutes p.i., C: 4h p.i.). Late HMR 1.63.
Copyright American Society of Nuclear Cardiology

## Slide 7
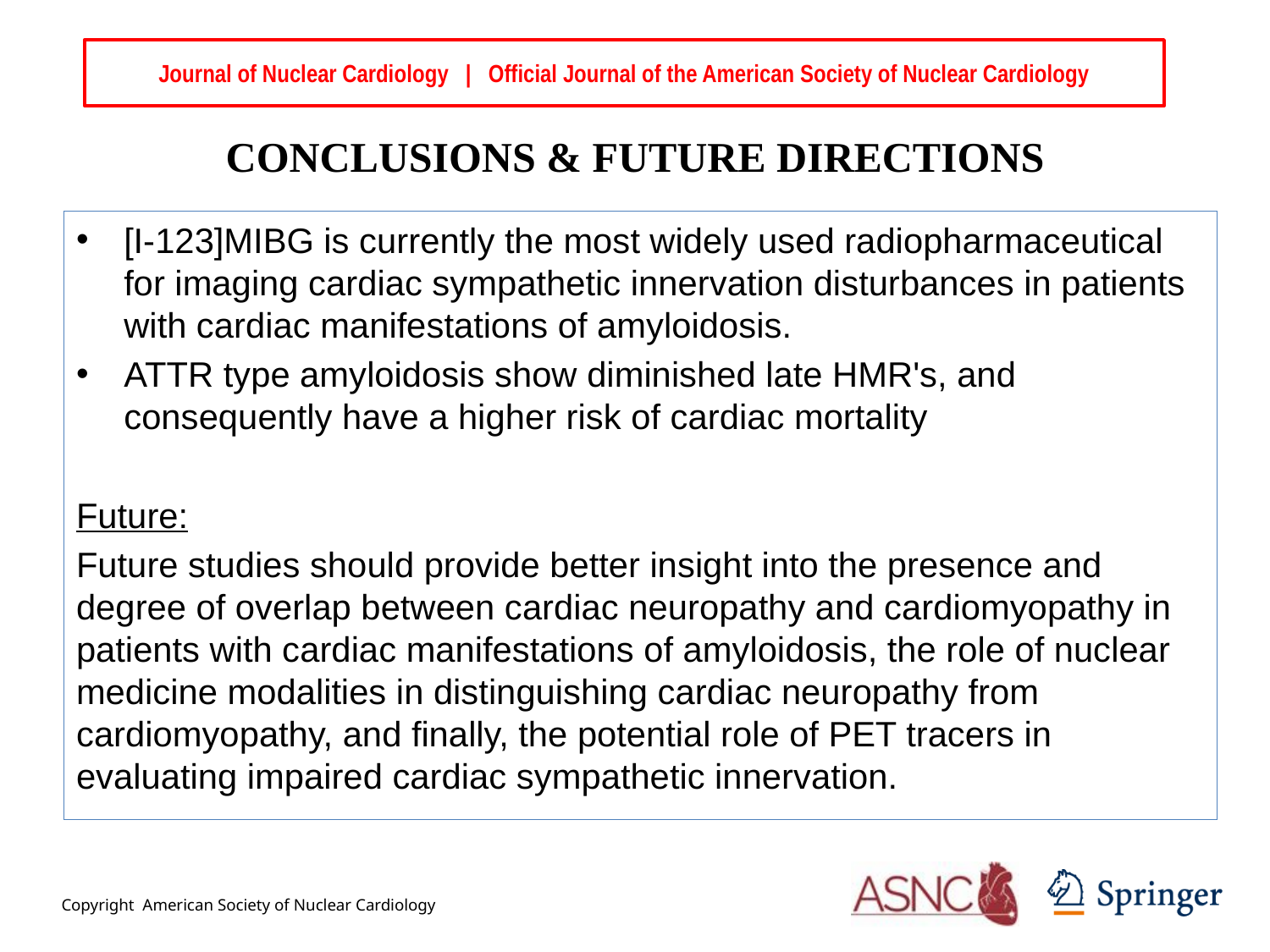

Journal of Nuclear Cardiology | Official Journal of the American Society of Nuclear Cardiology
# CONCLUSIONS & FUTURE DIRECTIONS
[I-123]MIBG is currently the most widely used radiopharmaceutical for imaging cardiac sympathetic innervation disturbances in patients with cardiac manifestations of amyloidosis.
ATTR type amyloidosis show diminished late HMR's, and consequently have a higher risk of cardiac mortality
Future:
Future studies should provide better insight into the presence and degree of overlap between cardiac neuropathy and cardiomyopathy in patients with cardiac manifestations of amyloidosis, the role of nuclear medicine modalities in distinguishing cardiac neuropathy from cardiomyopathy, and finally, the potential role of PET tracers in evaluating impaired cardiac sympathetic innervation.
Copyright American Society of Nuclear Cardiology
